# The first-line antituberculosis drugs, and their fixed-dose combination induced abnormal sperm morphology and histological lesions in the testicular cells of male mice

**DOI:** 10.3389/fcell.2022.1023413

**Published:** 2022-12-13

**Authors:** Adekunle A. Bakare, Victoria Y. Moses, Charles T. Beckely, Toluwani I. Oluyemi, Gift O. Ogunfeitimi, Aduragbemi A. Adelaja, Glory T. Ayorinde, Adeyinka M. Gbadebo, Olukunle S. Fagbenro, Olusegun I. Ogunsuyi, Opeoluwa M. Ogunsuyi, Olusoji Mayowa Ige

**Affiliations:** ^1^ Cell Biology and Genetics Unit, Department of Zoology, University of Ibadan, Ibadan, Nigeria; ^2^ Department of Biological Science, Chrisland University, Abeokuta, Nigeria; ^3^ Department of Biological Sciences, College of Basic and Applied Sciences, Mountain Top University, Ibafo, Ogun State, Nigeria; ^4^ Department of Cell Biology and Genetics, University of Lagos, Akoka, Lagos State, Nigeria; ^5^ Department of Medicine, Faculty of Clinical Sciences, College of Medicine, University of Ibadan, Ibadan, Nigeria

**Keywords:** first-line antituberculosis drug, DNA damage, ethambutol, isoniazid, rifampicin, pyrazinamide, sperm morphology

## Abstract

Rifampicin (RIF), Isoniazid (INH), Ethambutol (EMB), Pyrazinamide (PZA), and/or their fixed-dose combination (FDC) are extensively prescribed in the cure of Tuberculosis (TB) globally. In spite of the beneficial effect, these drugs are capable of inducing cellular toxicity. Existing information on the genotoxic effects of the first-line anti-TB drugs is limited and contentious. Herein, we evaluated the reproductive genotoxicity of RIF, INH, EMB, PZA, and their FDC utilizing the mouse sperm morphology assay. Histological examination of the testes of exposed mice was also performed. Male Swiss albino mice (11–13 weeks old) were intraperitoneally exposed for 5 consecutive days to each of the anti-TB drugs at four different doses of 6.25, 12.5, 25, and 50 mg/kg bw of PZA; 2.5, 5.0, 10, and 20 mg/kg bw of RIF; 1.25, 2.5, 5.0 and 10 mg/kg bw of INH; 3.75, 7.5, 15 and 30 mg/kg bw of EMB; and 7, 14, 28 and 56 mg/kg bw of FDC corresponding respectively to ×0.25, ×0.5, ×1 and ×2.0 of the standard daily dose. In comparison with the negative control (normal saline), there was no significant difference in the testicular weight and organo-somatic index of exposed mice. There was an increase (*p* > 0.05) in the frequency of abnormal spermatozoa at most of the tested doses of each drug and a dose-dependent decrease with the FDC. Each of the anti-TB drugs except the FDC induced pathological lesions in the testes. These findings suggest that the individual first-line anti-TB drug unlike the FDC has the potential to provoke testicular anomalies in male mice.

## Introduction

Tuberculosis (TB) is an infectious airborne disease caused in humans by the pathogenic bacteria, *Mycobacterium tuberculosis* (Anne-Laure et al., 2015). TB typically infects the lungs, but can also affect other parts of the body. It is one of the oldest recorded human diseases, which remains among the major health threats leading to morbidity and mortality globally (Raviglione and Sulis, 2016). According to the WHO (2021), 43% of all new TB cases in 2020 were found in the WHO South-East Asian Region followed by the WHO African Region, with 25%, and the WHO Western Pacific with 18%. After COVID-19, TB is the second foremost infectious killer and the 13th leading source of mortality worldwide. In Nigeria, TB is a serious public health problem (Ganiyu et al., 2021). The predicted burden of TB in Nigeria in 2019 was 440,000 TB cases; of these cases, 117,320 were diagnosed and notified to the National TB Programme (USAID 2021). The country ranked sixth among the 30 high TB burden countries and high multidrug-resistant TB burden countries (Global tuberculosis report, 2020).

Treatment of TB with antibiotics can be advantageous to lessen the spread to other persons, together with other public health measures, like isolation and cough etiquette. Generally, two different steps are involved in the treatment of tuberculosis: the intensive (bactericidal) phase and the continuation (sterilizing) phase. During the intensive phase, mycobacteria with a high replication rate are killed, while in the continuation phase, semi-dormant bacteria are eliminated (Sotgiu et al., 2015). For the duration of the 2-month intensive phase of treatment, the first-line standard regimen usually prescribed includes two bactericidal drugs [isoniazid (INH) with rifampicin (RIF)], ethambutol (EMB) to inhibit mono-resistant strains and to reduce the mycobacterial burden, and pyrazinamide (PZA), whose action is mainly focused to the semi-dormant mycobacteria. The adoption of a fixed-dose, single-tablet combination (FDC) is favored over separate drug formulations in the treatment of patients with drug-susceptible TB due to noncompliance (Zuur et al., 2016). In the intensive phase, the FDC involves the combination of RIF, INH, PZA, and EMB for 2 months followed by the continuation phase with the combination of INH and RIF for another 4 months. Combination chemotherapy usually produce positive outcomes through different mechanisms including synergistic, additive, and potentiation effects (Hu et al., 2015).

Anti-TB drugs are often well tolerated, but there may be toxic side effects in up to 80% of patients with TB (Arbex et al., 2010). The most common and dominant toxic effects of anti-TB drugs is hepatotoxicity associated with INH, RIF, and PZA (Steele et al., 1991; Jeong and Park, 2015). There has also been a report of EMB-induced liver cholestatic jaundice under unclear circumstances (Saukkonen et al., 2006). Multiple drug regimens, potentiate the adverse effects of antituberculosis therapy. Thus, though INH, RIF, and PZA each in itself are potentially hepatotoxic, when given in combination, their toxic effect is enhanced (Pandit et al., 2012). Adverse responses to anti-TB medications have been associated with the dosage, time of day at which the drug is administered, patient genotype, age, nutritional state, and the existence of pre-existing illnesses or dysfunctions, such as alcohol disorder, impaired kidney and liver function, and HIV co-infection (Javadi et al., 2007; Chung-Delgado et al., 2011; El Hamdouni et al., 2020).

Anti-TB drugs are pharmaceuticals of long-term clinical use, and genotoxic and mutagenic effects are among the detrimental effects these medications may cause. Studies on the genotoxic and mutagenic effects of first-line anti-TB drugs, singly and as a combination, are few (Masjedi et al., 2000; Brambilla et al., 2012; Fatima et al., 2013; Arslan et al., 2015). These medications have been shown to have synergistic and/or antagonistic effects when used as different regimen combinations, but no conclusive or consistent findings have been made. While some authors concluded that these medications, either alone or in combination, are not mutagenic, others demonstrated the anti-TB medications’ synergistic interaction in provoking genetic damage (Masjedi et al., 2000). These contradictions may partly reflect the usage of different bioassays and endpoints. We are not aware of report on the reproductive genotoxicity of anti-TB drugs in mice using the sperm morphology assay. Herein, we explored the genotoxic and reproductive toxicity of the first-line anti-TB drugs RIF, INH, PZA, and EMB singly and as an FDC using the mouse sperm morphology test. In addition, we checked the effects of the test drugs on testicular indices and histology.

## Materials and methods

### Animals

Healthy male Swiss albino mice (*Mus musculus*; 11–13 weeks old) acquired from the animal breeding facility of the Department of Zoology, University of Ibadan, Nigeria were kept in clear plastic cages coated with wood shavings and fed normal food pellets and water *ad libitum*. The normal day-night cycle, ambient temperature, and relative humidity were all applied to the animals. The mice were handled in accordance with the norms outlined in the Guidelines for the Use of Animals in Biomedical Research (CIOMS, 2012). The study was approved by the University of Ibadan’s Animal Care and Use Research Ethics Committee (UI-ACUREC; approval number: UI- ACUREC/18/0058).

### Test drugs and preparation

Ethambutol (400 mg), Isoniazid (300 mg), Pyrazinamide (500 mg), and Rifampicin (300 mg) were obtained commercially, while the FDC (900 mg of 150 mg RIF, 75 mg INH, 400 mg PZA and 275 mg EMB) was obtained from the Medical Out-Patient unit of the University College Hospital, Ibadan, Nigeria. The approved daily dose in TB treatment for adults consists of INH 5 mg/kg; RIF 10 mg/kg; PZA 25 mg/kg; EMB 15 mg/kg and the FDC 28 mg/kg, dependent on body weight (World Health Organization, 2010; Zuur et al., 2016). The tested doses of each of the drugs and the FDC were prepared from a stock solution of each drug considering the approved daily dose, and body weight (bw) of the experimental animals. Four doses: ×0.25, ×0.5, ×1 and ×2.0 of the standard daily dose corresponding respectively to 6.25, 12.5, 25 and 50 mg/kg bw of PZA; 2.5, 5.0, 10, and 20 mg/kg bw of RIF; 1.25, 2.5, 5.0 and 10 mg/kg bw of INH; 3.75, 7.5, 15 and 30 mg/kg bw of EMB; and 7, 14, 28 and 56 mg/kg bw of FDC (Table 1) were considered. The administration and duration of animal exposure were carried out in accordance with standard protocols (Wyrobek et al., 1983; Ogunsuyi et al., 2020; Oyinleye et al., 2021).

**TABLE 1:** Experimental plan on the dose regimen of each of the first-line antituberculosis drugs and their fixed-dose combination.

Drugs	Doses (mg/kg bw)			
	×0.25	×0.5	×1	×2
Pyrazinamide	6.25	12.50	25.00	50.00
Rifampicin	2.50	5.00	10.00	20.00
Ethambutol	3.75	7.50	15.00	30.00
Isoniazid	1.25	2.50	5.00	10.00
Fixed-dose combination	7.00	14.00	28.00	56.00

### Experimental design and sperm morphology assessment

The design of Wyrobek et al. (1983), with minor adjustment (Ogunsuyi et al., 2020; Oyinleye et al., 2021) was utilized. There were 22 groups of mice [*n* = 8 (5 for the sperm morphology assay and 3 for the testes weight and histopathology) (Table 1)]. Due to mortality of experimental mice during the study period, four additional groups of three mice each were added to each of the NC and PC for each drug respectively, for the assessment of testes weight and histopathology. This thus increased the replicates of each control group to five (NC = Groups 21, 21a, 21b, 21c, and 21d; PC = Groups 22, 22a, 22b, 22c, and 22d). For each of the drugs or the FDC, mice were randomly divided into four groups corresponding to the four doses (Table 1). Respectively, groups 1–4 received 6.25, 12.5, 25 and 50 mg/kg bw of PZA; groups 5–8 received 2.5, 5.0, 10, and 20 mg/kg bw of RIF; groups 9–12 received 3.75, 7.5, 15 and 30 mg/kg bw of EMB; groups 13–16 received 1.25, 2.5, 5.0 and 10 mg/kg bw of INH; and groups 17–20 received 7, 14, 28 and 56 mg/kg bw of the FDC. Each group was administered *via* the intraperitoneal (IP) route with 0.5 ml of the respective dose for five consecutive days. Groups 21 (and 21a - d), and 22 (and 22a - d) received IP 0.5 mL each of normal saline and cyclophosphamide (20 mg/kg bw) as negative (NC) and positive (PC) controls, respectively for five consecutive days. Mice in all the groups were maintained for another 30 days and were sacrificed by cervical dislocation at day 35 from the first day of exposure, after which their cauda epididymides and testes surgically excised. The exposure duration of 35 days was selected based on the completion of mouse spermatogenesis, which is 35 days (Bartke et al., 1974; Hess and Chen, 1992). The cauda epididymis of each testis was then minced in normal saline and stained with 1% Eosin Y to create sperm smears on glass slides. The slides were thereafter air-dried and arranged in groups for the subsequent microscopic examination at ×1000. One thousand spermatozoa from each mouse were examined for morphological abnormalities (Ogunsuyi et al., 2020; Oyinleye et al., 2021).

### Relative organ weight of the testes and histopathology

On the first and last day of the experiment, the body weight of each mouse in each group was recorded. Testes were preserved in Bouin’s solution after being washed in physiological saline (pH = 7.4), blotted with filter paper, and weighed. In accordance with Oyeyemi et al. (2017), sections of the testes were prepared and processed for histological examination at ×200 and ×400. The organo-somatic index, or relative organ weight, was determined as follows:

$$Relative\;Organ\;Weight=\frac{Absolute\;organ\;weight\;\left(g\right)x100}{Final\;body\;weight\;of\;animal\;prior\;to\;sacrifice\;\left(g\right)}$$

### Analysis of data

GraphPad Prism 5.0 statistical software was used for data analysis. Data were presented as absolute values, percentage frequency and mean ± standard error using One-way ANOVA with Dunnett post-hoc test to detect differences among groups. Mean values of test groups were compared with negative control (NC) to determine the level of significance. Differences between compared groups were considered statistically significant at *p* < 0.05.

## Results

### Testicular weight, organo-somatic index and histopathology

**FIGURE 1:**
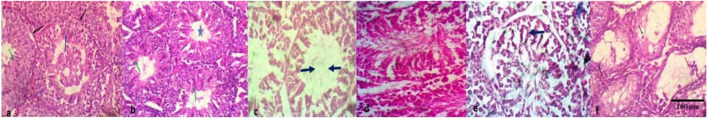
Histopathologic lesions in testes of mice treated with the first-line antituberculosis drugs: rifampicin, isoniazid, pyrazinamide, and ethambutol, and their fixed-dose combination. **(A)** no visible lesion; seminiferous tubules with regular outlines (black arrow) having predominant stages of spermatocytes (blue arrow) and elongated spermatids, **(B)** moderately- sized seminiferous tubule with no lesion, and markedly-densed spermatids (green arrow) in its lumen (star), **(C)** seminiferous tubules with degeneration of Sertoli cells, loss of the germinal epithelium and increase in luminal width, **(D)** Seminiferous tubules with patchy loss of germinal epithelium, retention of spermatids and varied sizes of seminiferous tubules, **(E)** seminiferous tubules with degeneration of Sertoli cells, retention of spermatids and syncytial formation of degenerate spermatids (black arrow), **(F)** seminiferous tubule with necrotic spermatogenic cells (black arrow) and mildly-densed spermatocytes (red) in the lumen. Magnification: ×200–400.

Compared to the NC, there was no significant (*p* > 0.05) difference in the testicular weight and organo-somatic index of mice treated with the individual first-line anti-TB drugs and the FDC except at the 20 mg/kg bw of RIF and 7.5 mg/kg bw of EMB (Table 2). Normal tissue cellular arrangement was observed in the testes of mice in the NC group (Figures 1A,B), but in the groups treated with each of the first-line anti-TB drugs, there were histological abrasions such as irregular seminiferous tubule outlines, reduction of seminiferous epithelium, increase in luminal width, necrotic spermatogenic cells, congestion of the interstitial blood vessels, and degeneration of Sertoli cells (Figures 1C–F). There was no lesion in the testes of mice exposed to the FDC.

**TABLE 2:** Testicular indices in mice exposed to antituberculosis drugs.

Dose (xSDD)	PZA		RIF		EMB		INH		FDC	
	Absolute testes weight (g)	Relative testes weight (%)	Absolute testes weight (g)	Relative testes weight (%)	Absolute testes weight (g)	Relative testes weight (%)	Absolute testes weight (g)	Relative testes weight (%)	Absolute testes weight (g)	Relative testes weight (%)
NC	0.18 **±** 0.03	0.57 **±** 0.10	0.07 **±** 0.01	0.29 **±** 0.01	0.08 **±** 0.007	0.29 **±** 0.03	0.21 **±** 0.01	0.74 **±** 0.05	0.21 **±** 0.01	0.85 **±** 0.04
0.25	0.19 ± 0.01	0.65 ± 0.03	0.10 ± 0.01	0.37 ± 0.03	0.09 **±** 0.007	0.35 **±** 0.02	0.16 ± 0.01*	0.61 ± 0.05	0.17 **±** 0.02	0.71 **±** 0.06
0.5	0.20 ± 0.01	0.71 ± 0.06	0.10 ± 0.00	0.35 ± 0.01	0.10 **±** 0.004	0.39 **±** 0.02*	0.20 ± 0.01	0.74 ± 0.03	0.24 **±** 0.01	0.87 **±** 0.05
1.0	0.23 ± 0.01	0.74 ± 0.07	0.06 ± 0.01	0.23 ± 0.02	0.07 **±** 0.005	0.26 **±** 0.02	0.21 ± 0.02	0.66 ± 0.05	0.20 **±** 0.01	0.74 **±** 0.03
2.0	0.21 ± 0.01	0.70 ± 0.03	0.09 ± 0.01	0.38 ± 0.04*	0.08 **±** 0.004	0.30 **±** 0.01	0.23 ± 0.01	0.76 ± 0.06	0.22 **±** 0.01	0.84 **±** 0.04
PC	0.19 **±** 0.01	0.64 **±** 0.03	0.06 **±** 0.01	0.27 **±** 0.05	0.06 **±** 0.010	0.27 **±** 0.05	0.18 **±** 0.01	0.71 **±** 0.03	0.19 **±** 0.01	0.74 **±** 0.04

All values are x̄ ± S.E. *p < 0.05 significant when compared with the negative control. SDD, standard daily dose; NC, negative control (normal saline); PC, positive control (cyclophosphamide, 20 mg/kg); PZA, pyrazinamide; RIF, rifampicin; EMB, ethambutol; INH, isoniazid; FDC, full dose combination.

### Analysis of sperm abnormalities

**FIGURE 2:**
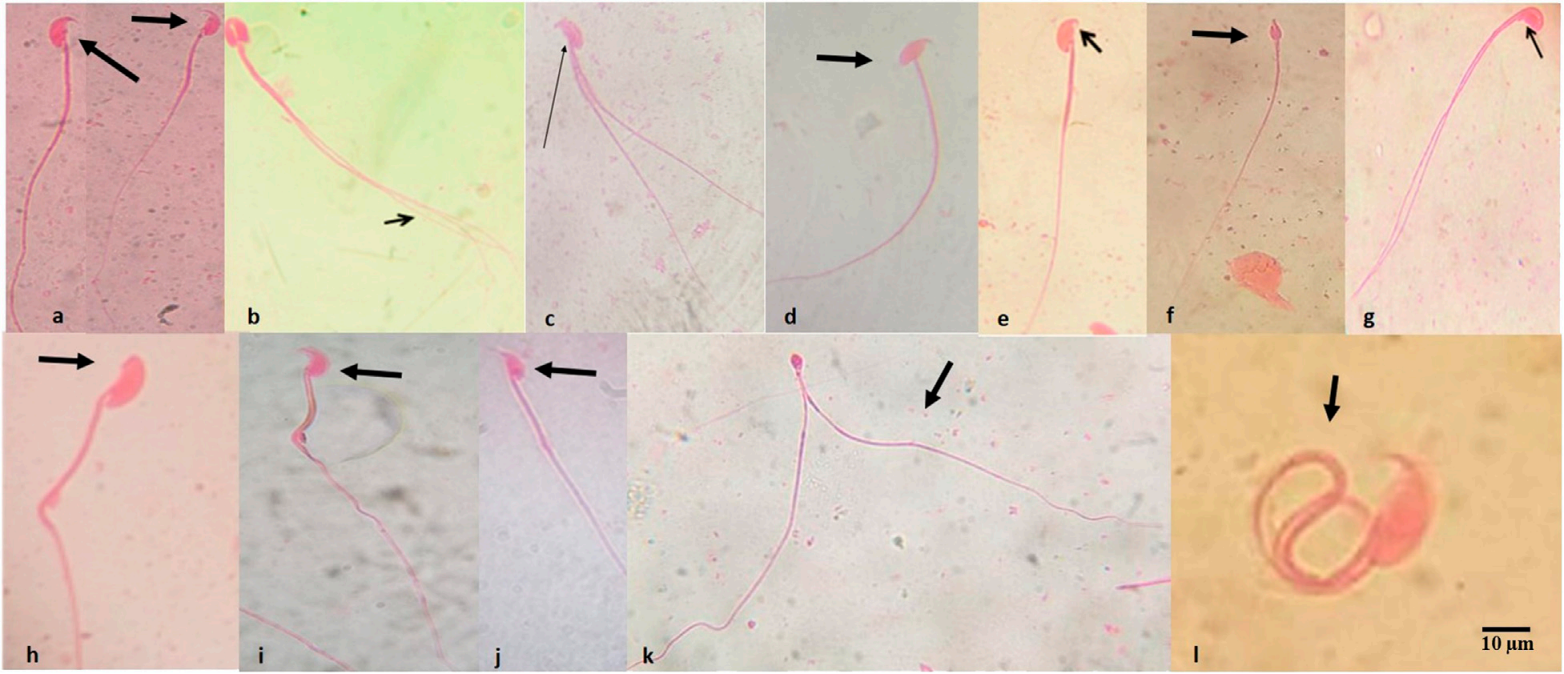
Abnormal spermatozoa induced by the first-line antituberculosis drugs: rifampicin, isoniazid, pyrazinamide, and ethambutol, and their fixed-dose combination in mice. **(A)** Normal spermatozoa, **(B)** fused head with double tails, **(C)** double tail with short hook, **(D)** wrong-tail attachment, **(E)** knobbed hook, **(F)** amorphous head, **(G)** amorphous head with double tails, **(H)** no hook, **(I)** banana head, **(J)** hook at wrong angle, **(K)** sperm cells with pin head and double tails, **(L)** folded sperm cell. Magnification: ×1000.

Table 3 presents the summary of the frequency of abnormal spermatozoa induced in mice treated with the first-line anti-TB drugs and their FDC. Compared to the NC, there was a nonsignificant (*p* > 0.05) increase in the number of abnormal spermatozoa (Figures 2A–L) at each of the tested doses of each drug and the FDC, except at the 50 mg/kg bw (x2) of PZA, 5.0 mg/kg bw (x0.5) of RIF, 7.5 mg/kg bw (x0.5) of EMB, and 28 mg/kg bw (x1) and 56 mg/kg bw (x2) of the FDC (Table 3). Only mice exposed to the FDC exhibited a dose-dependent decrease in the frequency of abnormal spermatozoa. The positive control induced a statistically significant (*p* < 0.05) increase in the frequency of abnormal spermatozoa. The sperm abnormalities induced by the first-line anti-TB drugs and their FDC include spermatozoa with a short hook, no hook, folded sperm, wrong tail attachment, wrong-angled hook, knobbed hook, fused sperm cells, amorphous head, banana head, pinhead, double tail, three-tailed and sperm with double head (Figures 2B–L).

**TABLE 3:** Summary of abnormal spermatozoa produced in the testes of mice treated with different doses of the first-line antituberculosis drugs and the fixed-dose combination.

Dose (xSDD)	PZA			RIF			EMB			INH			FDC		
	Total abnormal spermatozoa^a^	Mean	Frequency (%)^b^	Total abnormal spermatozoa^a^	Mean	Frequency (%)^b^	Total abnormal spermatozoa^a^	Mean	Frequency (%)^b^	Total abnormal spermatozoa^a^	Mean	Frequency (%)^b^	Total abnormal spermatozoa^a^	Mean	Frequency (%)^b^
NC	296	32.9	5.9	296	32.9	5.9	296	32.9	5.9	296	32.9	5.9	296	32.9	5.9
0.25	493	41.0	9.9	378	37.8	7.6	389	35.4	7.8	608	60.8	12.2	420	42.0	8.4
0.5	561	46.8	11.2	196	21.8	3.9	255	25.5	5.1	466	51.8	9.3	343	34.3	6.9
1.0	479	36.9	9.6	636	70.7	12.7	315	35.0	6.3	417	46.3	8.3	252	28.0	5.0
2.0	201	15.4	4.0	612	68.0	12.2	490	49.0	9.8	513	46.6	10.3	250	25.0	5.0
PC	1006	91.5*	20.1	1006	91.5*	20.1	1006	91.5*	20.1	1006	91.5*	20.1	1006	91.5*	20.1

^a^ Scored based on 1,000 sperm cells/mouse and five mice/group.

^b^ % frequency of abnormal spermatozoa based on total aberrant cells.

Differences between groups were considered statistically significant at *p < 0.05 when compared to the negative control. SDD, standard daily dose; NC, negative control (normal saline); PC, positive control (cyclophosphamide, 20 mg/kg); PZA, pyrazinamide; RIF, rifampicin; EMB, ethambutol; INH, isoniazid; FDC, full dose combination.

## Discussion

A significant stage in the origin of many genetic disorders, birth abnormalities, and somatic diseases like cancer is the activation of DNA damage. In 2004, the WHO established the first global plan on this issue and declared the protection of reproductive health as a global priority. The possibility of a large variety of xenobiotics (including medicines) to alter the function of the male reproductive system was given particular attention among the possible threats to reproductive health. Currently, it is understood that using anti-TB medications concurrently and for an extended period of time may have detrimental effects on how quickly proteins and amino acids are metabolized (Kovalenko et al., 2007; Bondarenko et al., 2011). The epidemiological situation of TB keeps worsening, yet there is little information on the harmful side effects of anti-TB medications, particularly those that may affect the reproductive system. Hence, this study on the appraisal of the reproductive toxicity of RIF, INH, PZA, and EMB, and their FDC in male mice.

The evaluation of organ weight is a sensitive indicator for the assessment of the effect of chemicals on organs (Michael et al., 2007); with an increase or decrease in testicular weight being a useful tool for evaluating toxicity that affects spermatogenesis (Desai et al., 2016). Testicular weights bring to light sensitivity to disruptions in swiftly proliferating cells, physiology, and hormones. They also assist to spot enzyme induction and connect well with histological modifications (Michael et al., 2007). In this study, none of the tested drugs caused significant changes in the relative testicular indices when compared with the NC, except at the 20 mg/kg bw (x2) of RIF and 7.5 mg/kg bw (x0.5) of EMB which showed a significant increase in the testicular weight. An increase in testicular weight usually reflects an increase in seminiferous tubular fluid content and is usually accompanied by an increase in the diameter of the tubular lumen. However, the overall observation implied that the test drugs did not adversely affect the growth of the mice testes.

Analysis of histological parameters has proven to be a reliable and sensitive biomarker and may help indicate the health status of the experimental animal being studied (Iyiola et al., 2017). Necrotic spermatogenic sperm cells, increased luminal width, and interstitial blood vessels were observed in mice exposed to the individual anti-TB drugs except for the FDC that showed no testicular lesions. Congestion of blood vessels suggests that the anti-TB drugs may have impaired venous outflow in the mice testes and thus, resulted in congestion. Awodele et al. (2016) attributed decreased sperm counts and motility to choked vessels in the peri-testicular and Leydig cells of the stroma in the testes of rats exposed to the FDC and was linked to a reduction in male fertility. Testicular degeneration involves a retrogressive change in the germinal epithelium of the seminiferous tubules (Macfarlane et al., 2001). The retention of spermatids in the seminiferous tubules is as a result of disruption of spermiation which causes the continued presence of the mature spermatids, thereby resulting in reduced epididymal sperm count, sperm abnormalities, and potential effects on fertility. Some of these changes are in concert with the findings of Shayakmetova et al. (2016) where morphological and morphometric changes were observed in the spermatogenic epithelium of rats with co-administered anti-TB drugs. Ethambutol and INH were found to induce spermatogenic epithelial abnormalities, obvious regressive lesions, and loss of fertility (Braun et al., 1984; Yue et al., 2009).

In the sperm morphology test, the development of sperm head anomalies has been used as a dependable short-term biomarker in the assessment of xenobiotics-induced genetic damage (Rasgele, 2014; Ogunsuyi et al., 2020). Across all dose levels, the test drugs caused an insignificant increase in the number of abnormal spermatozoa in mice compared to those in the NC group. This increase was, however, not observed at 50 mg/kg bw of PZA, 5.0 mg/kg bw of RIF, and 7.5 mg/kg bw of EMB, but there was a dose-dependent decrease in the number of aberrant sperm cells induced by INH at the 1.25–5 mg/kg bw, and the FDC. It is possible that INH, a substance with a weak direct genetic activity (Braun et al., 1984), contributed to the dose-dependent decrease of sperm abnormality at the dose range of 1.25–5 mg/kg bw in the FDC. The interaction of the four anti-TB drugs in the FDC could also have probably led to disruption of the process of spermatogenesis as the dose level increases, resulting in a dose-dependent decrease in the number of sperm cells. Considering the overall frequency (%) of induction of abnormal spermatozoa in mice, the order is INH (10.02%) > RIF (9.11%) > PZA (8.67%) > EMB (7.25%) > FDC (6.33). These indicate that the test drugs though not significant had an effect on sperm cells that had developed from treated spermatogonia cells. None of the test drugs produced any distinct type of aberrant spermatozoon but rather a variety of anomaly shaped sperm cells. The dominant types of sperm shape abnormalities viewed were those with folded shape, amorphous head, no hook, wrong-angled hook, and short hook.

The specific reason for the increasing frequency of abnormal sperm cells is not apparent, and opinions differ on this matter. Minor modification in testicular DNA, point mutations, and gross mutations occur during the arrangement of DNA in the sperm head were proposed as possible genetic causes of abnormal spermatozoa (Bruce et al., 1974; Wyrobek et al., 1983; Mendoza-Lujambio et al., 2002). The abnormalities may also arise due to mistakes in the spermatozoa-differentiating process during spermatogenesis (Wyrobek et al., 1983). Sperm morphological abnormalities are thus postulated to occur during spermatogenesis since sperm shape is quite stable once the sperm has developed.

The observation of the effect of the FDC is similar to those of Awodele et al. (2016) wherein they observed alteration of reproductive hormones in male and female rats when compared to the NC groups. Rifampicin is associated with hepatotoxicity mediated by oxidative damage (Shanker et al., 2005; Chowdhury et al., 2006). It was also shown to cause an increase in DNA oxidative damage in the liver of male mice (Safaa et al., 2013), a dose- and time-dependent increase in the percentage of chromosome aberrations and sister chromatid exchanges in mouse bone marrow (Aboul-Ela, 1995; Aly and Donya, 2002), and induction of micronuclei and comet in blood and kidney samples of rat (Arslan et al., 2015). Isoniazid is a promutagen with a weak direct genetic activity, which needs to undergo some metabolic activities in order to be activated as a mutagen. The genotoxic properties of INH in mammals are essentially determined by the pharmacokinetic behavior of the terminal reactive metabolite (Braun et al., 1984). Anitha et al. (1994) reported that PZA exhibited weak genotoxic effect on mice, but Arslan et al. (2015) showed that PZA had strong hepatotoxic effects and caused DNA damage in rats. ETA has less toxic effects on organisms than other anti-TB drugs (Bruton et al., 2009). According to Arslan et al. (2015), ETA caused DNA damage in blood, liver, and kidney, and increased the number of micronucleated cells in the blood of rats.

The histological and genotoxic changes in the testicular cells of the treated mice could have been due to the ability of metabolites of the anti-TB medications to cross the blood testes barrier (Shayakhmetova et al., 2012; Awodele et al., 2016). Apart from the possible action of each of the individual drugs, and their metabolites, it may be assumed that oxidative stress is part of the causative mechanisms (Shayakhmetova et al., 2012; Shayakhmetova et al., 2016). In the testicular tissues of mice exposed to the FDC, Shayakhmetova et al. (2016) hypothesized that the xanthine oxidase ROS-generating system was involved, and that the chelation of zinc and copper contributed to an imbalance of oxidative processes and antioxidant defence. The genotoxic effects of RIF and INH can be linked to CYP2E1 activation (Nishimura et al., 2004; Shayakmetova et al., 2012). PZA may act as a Cytochrome P450-2E1 inducer (Bondarenko et al., 2011). EMB may induce its toxic changes through the interaction with mitochondrial cytochrome oxidase (complex IV) activity through a copper-chelating action. It causes the extensive formation of heterogeneous vacuoles correlating with a decrease in mitochondrial membrane potential (Guillet et al., 2010). EMB is a metal chelator and may disrupt oxidative phosphorylation and mitochondrial function by interfering with iron-containing complex I and copper-containing complex IV. The interference of EMB with these complexes may result in an increase in the production of reactive oxygen species (Alonso et al., 2004).

Superoxide radicals, which are produced by CYP 2E1, are reactive oxygen intermediates that may quickly combine with organic molecules to produce secondary free radicals and reactive oxygen radical species (Lieber, 1997). Such cascades may alter the reducing ambience of testes, generating conditions for sperm (DNA) oxidative damage, which leads to DNA fragmentation, and cell death (Shayakhmetova et al., 2012). The discharge of spermatozoa from the germinal epithelium with unusually high levels of cytoplasmic retention and poorly modified chromatin is frequently caused by errors in spermiogenesis that arose from excessive free radical generation (Sanocka and Kurpisz, 2004). Since oxidative stress can cause serious disruption in nucleic acids and protein metabolism, structure, and functions and may play a significant role in inducing DNA disruption in germ cells, the death of spermatogenic cells in mice treated with INH may be attributable to intensified germ cell apoptosis brought on by oxidative stress stimulation (Aitken et al., 2014; Shayakmetova et al., 2016).

Overall, there seems to be a correlation between the testicular histology findings and the frequency of sperm abnormalities.

## Conclusion

This study has shown the interaction of Rifampicin, Isoniazid, Pyrazinamide, and Ethambutol in the fixed-dose combination to reduce the frequency of abnormal spermatozoa and cause no histological lesion in the testicular tissue. It has been established that combinatorial therapy is given for the treatment of tuberculosis and when these first-line drugs are given in combination, toxicity is strengthened. Our findings with the FDC suggest a reduction of toxicity in the testicular cells of exposed mice. Further studies at the pharmacokinetic level to evaluate the interactive toxicological effect of the individual drugs in the FDC is needed. Our data also indicate that Rifampicin, Isoniazid, Pyrazinamide, and Ethambutol individually may have a genotoxic potential in the germ cells of male mice when exposed for a long period of time. Tuberculosis continue to be one of the world’s fatal contagious ill health (Zuur et al., 2016). Our findings are of relevance in clinical studies because most TB patients rely on the first-line anti-TB drugs for their survival. Studies on the assessment of somatic genotoxic effects of the anti-TB drugs are in progress.

## Data Availability

The original contributions presented in the study are included in the article, further inquiries can be directed to the corresponding author.
